# Emerging Persistent Organic Pollutants in Chinese Bohai Sea and Its Coastal Regions

**DOI:** 10.1155/2014/608231

**Published:** 2014-02-03

**Authors:** Xiaomin Li, Yan Gao, Yawei Wang, Yuanyuan Pan

**Affiliations:** ^1^Institute of Quality Standard and Testing Technology for Agro-Products, The Chinese Academy of Agricultural Sciences (CAAS), Beijing 100081, China; ^2^State Key Laboratory of Environmental Chemistry and Ecotoxicology, Research Center for Eco-Environmental Sciences, Chinese Academy of Sciences, Beijing 100085, China; ^3^Thermo Fisher Scientific, Room 214, Tianchuang Science and Technology Building, No. 8 Caihefang Road, Haidian District, Beijing 100080, China

## Abstract

Emerging persistent organic pollutants (POPs) have widely aroused public concern in recent years. Polybrominated diphenyl ethers (PBDEs) and perfluorooctane sulfonyl fluoride/perfluorooctane sulfonic acid (POSF/PFOS) had been newly listed in Stockholm Convention in 2009, and short chain chlorinated paraffins (SCCPs) and hexabromocyclododecanes (HBCDs) were listed as candidate POPs. Bohai Sea is located in the arms of numbers of industrial cities, the semienclosed location of which makes it an ideal sink of emerging pollutants. In the present paper, latest contamination status of emerging POPs in Bohai Sea was reviewed. According to the literature data, Bohai Sea areas are not heavily contaminated by emerging POPs (PBDE: 0.01–720 ng/g; perfluorinated compounds: 0.1–304 ng/g; SCCPs: 64.9–5510 ng/g; HBCDs: nd-634 ng/g). Therefore, humans are not likely to be under serious risk of emerging POPs exposure through consuming seafood from Bohai Sea. However, the ubiquitous occurrence of emerging POPs in Bohai Sea region might indicate that more work should be done to expand the knowledge about potential risk of emerging POPs pollution.

## 1. Introduction

A dozen of organic pollutants, including eight types of organochlorine pesticides (OCPs), two types of industrial chemicals (polychlorinated biphenyls (PCBs) and hexachlorobenzene (HCB)), and two types of byproducts (polychlorinated dibenzo-p-dioxins (PCDD) and polychlorinated dibenzofurans (PCDF), have been restricted as “legacy persistent organic pollutants (POPs)” by the Stockholm Convention in 2001. Due to POPs' physical and chemical properties, they are (1) environmental persistent; (2) widely distributed throughout the environment; (3) accumulate through food chain including humans; (4) toxic to humans and wildlife [1]. Stockholm Convention is open to compounds which have met the screening criteria for POPs. Emerging contaminants are presently referred to as pharmaceuticals and personal-care products, drugs of abuse, steroids and hormones, surfactants, flame retardants, industrial additives and agents, and so on [2–4], among which polybromodiphenyl ether (PBDEs), perfluorinated compounds (PFCs), short-chained chlorinated paraffins (SCCPs), and hexabromocyclododecane (HBCDs) were the most concerned. With the process of the work of POPs review committee, nine types emerging organic pollutants including perfluorooctane sulfonic acid (PFOS), commercial pentabromodiphenyl ether (penta-BDE), and commercial octabromodiphenyl ether (octa-BDE) have already been added to Stockholm Convention in 2009. Recently, SCCPs, HBCDs and other three emerging contaminants were proposed for being listed under the Convention.

China is a big country in producing and using chemicals, and the pollution problem of legacy POPs has continuously attracted public attention. Candidate POPs, also called “emerging POPs,” have become a hotspot in the environmental research and management.

**Figure 1:**
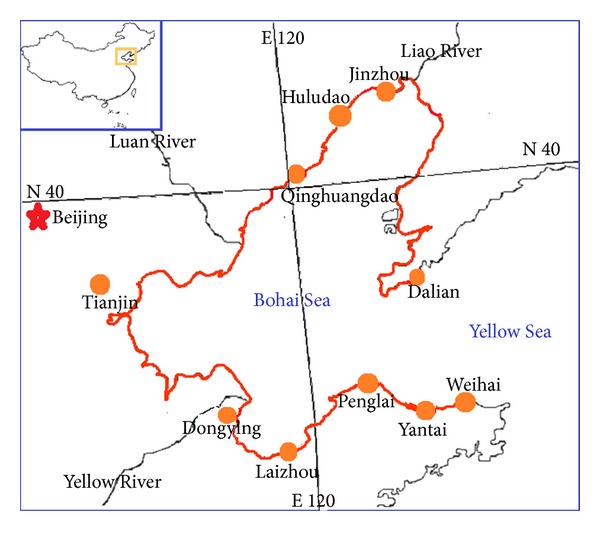
Main cities and rivers around Bohai Sea.

Chinese Bohai Sea is located within the arms of Liaodong and Shandong Peninsula (Figure 1) and is considered to be one of the most POPs polluted areas in the world. As one of the most developed regions in China, it is embraced by several metropolises, such as Beijing, Tianjin, and Dalian. Several rivers empty into Bohai Sea, meaning that the industrial and municipal wastewater from the surrounding cities is also discharged into it. In addition, the semienclosed terrain makes the water exchange between Bohai Sea and the open ocean relatively slow. Thus, pollutants in Bohai Sea are difficult to diffuse. Bohai Sea has become one of the most heavily polluted sea areas in China at present and might act as a sink of many pollutants including POPs [5–7]. However, Bohai Sea is one of the most important fisheries in China. It provides amounts of seafood to peripheral cities. Therefore the pollution in Bohai Sea had caused great concerns in the past decades. Many studies have focused on the pollution status of various chemicals such as heavy metals, organic metals, OCPs, and PCBs [5, 8–11]. As a very good bioindicator, a series of works of pollutants in mollusks along the coastline of the Bohai Sea has been reported since 2000s. Liang et al. [5] evaluated mollusks as biomonitors to investigate heavy metal contaminations along the Chinese Bohai Sea in 2004; Wang et al. [10] further studied the correlations among heavy metals in mollusks samples. The speciation of several heavy metals in mollusks such as mercury and tin was also studied. Liang et al. [8] evaluated the methylmercury and total mercury contents in gastropod and bivalve species from Chinese Bohai Sea. Yang et al. [12, 13] studied the distribution and temporal trends of butyltins monitored by mollusks along the Chinese Bohai coast and found that the concentrations of the concentration of butyltins kept high in mollusks during the sampling period of 2002~2005.

Some emerging pollutants such as PBDEs, SCCPs, and PFCs have been proved to be global pollutants. They were not only found in industrial areas but were also detected in remote areas such as Polar Regions and Tibet Plateau. This review have summarized and analyzed monitoring data available of main emerging POPs in Bohai Sea from peer-reviewed papers. Though these pollutants were analyzed by different analytical methods in various environmental media, the contamination status of emerging POPs and relative levels among legacy POPs and emerging POPs still should be clarified.

## 2. Analytical Methodologies

Emerging POPs usually exist at trace or ultratrace levels in environment; thus specific pretreatment and sensitive detection instrument are indispensable. According to the currently reported analytical methods for the most frequently studied emerging POPs, mass spectrometric detector was widely applied. Generally, chemicals with high volatility and thermal stability such as PBDEs and SCCPs were usually detected by GC-MS^n^, and those with high water solubility and/or polarity such as PFCs and HBCDs were analyzed by HPLC-MS [2].

## 3. Distributions of Emerging POPs in Water Body and Sediments

Sediment is viewed as an important sink of all the pollutants. Sediment contamination is essential in assessing the impact of human activities on aquatic systems. Pollutants which are released into water body will reach a dynamic balance between water phase and sediment. Besides, benthonic organisms such as mollusks can accumulate toxicants and introduce them to the food web from sediment and water phase; thus sediment plays a significant role in evaluating the overall environmental quality of an aquatic system.

### 3.1. PBDEs

PBDEs were additive brominated flame retardants which have been widely used around the world. Wang et al. [14] investigated congener profiles and pollution levels of PBDEs in sediment along the coast of Bohai Sea. The median values of BDE 209 and total PBDEs (including BDE 17, 28, 47, 66, 71, 85, 99, 100, 138, 153, 154, 183, and 190) were 2290 and 160 pg/g dry weight (dw) in sediments. Clearly, BDE 209 was the predominant congener in sediments. Pan et al. [15] examined PBDEs in 44 surface sediments from Bohai Sea. It has been found that the pollution level of seven major PBDEs (BDE 28, 47, 99, 100, 153, 154, and 183) ranged from 220 to 900 pg/g dw with an average of 480 pg/g dw. This is comparable to another semiclosed sea [16]. They also observed an average BDE 209 concentration of 7000 pg/g dw, which was one order of magnitude higher than the other BDE congeners. Besides, high levels of PBDEs were detected in sediments from river estuaries, which proved that the input generated by anthropogenic activities is one of the main sources of PBDEs in Bohai Sea. Further, Pan et al. studied the PBDE profiles in riverine and marine sediments of the Laizhou Bay. It is located in the southern Bohai Sea [17] and considered the largest manufacturing base of brominated flame retardants in Asia. The results showed that BDE 209 and the sum of other seven PBDE congeners in marine sediments ranged from 660 to 12000 pg/g and not detected (nd) to 660 pg/g, respectively. PBDEs contents in riverine sediments were higher than those in marine sediments, which might suggest the contributions of BFR manufacturing base. However, concentrations of PBDEs in both riverine and marine sediments were not at a peak value around the world. Zheng et al. [18] studied the PBDEs levels and profiles in the main riverine, estuarine, and intertidal zone sediments along the Bohai Bay coastline in 2007. PBDEs concentration in sediments ranged from nd to 870 pg/g dw. Similar to previous studies, higher levels of PBDEs were found in the sediments at the outlet of Haihe, Dagu Drainage Rivers, and Yongdingxinhe River. These results suggested that surface runoff might be one of the main introducing pathways of PBDEs into Bohai Sea. Zhao et al. [19] studied PBDEs distributions in three intertidal sediments (Qikou, Lujuhe, and Dagukou) according to grain size. No significant difference was found among fractions. The total PBDEs in sediment ranged from 56 to 300 pg/g dw, which were slightly lower than Pan et al.'s [15] study. Zhao et al. [20] investigated the concentrations, profiles, and possible sources of PBDEs in Daliao River estuary on north Bohai Sea coastline. The level of PBDEs in sediments was within the ranges of above studies. All the above studies confirmed that BDE 209 was the dominant congener in sediments, which indicated that commercial deca-BDE product might be the main source in Bohai Sea area, and the use of penta- and octa- BDE contained products that also exist.

### 3.2. PFCs

PFCs, especially PFOS and perfluorooctanoic acid (PFOA), have received considerable attention due to their persistence and ubiquitous existence in environment. Up to now, the information about PFCs distribution in the sediments from Bohai Sea is still limited, but several papers reported present PFCs distribution in waters and sediments of the rivers flowing to Bohai Sea. Chen et al. [21] investigated 15 perfluorinated compounds in water samples and corresponding surface sediments from outlets of rivers in coastal regions of Bohai Sea and found that PFOS and PFOA were the predominant PFCs in water samples. Compared to the reports of other places, total PFCs concentrations in most samples in this area were relatively low and the highest levels of PFOS and PFOA were, respectively, 31 and 82 ng/L. According to the geographical distribution of city and industry in their sampling areas, Chen et al. attributed the high levels of PFCs in some sampling sites to the industrial wastewater and domestic sewage discharge from cities, which was consistent with the findings by Becker et al. [22]. Generally, PFCs in sediment samples are higher than in water samples, and PFUnA is detected more frequently in sediment than PFOS and PFOA, which might indicate that sediment is a sink of long carbon chain PFCs. Wang et al. studied PFCs profiles in main riverine and estuarine around Bohai Sea area [23, 24]. PFCs levels in Liaohe River were significantly higher than other locations in North Bohai Sea coastline. Specifically, the PFCs level in water samples were consistent with Chen et al.'s study [21], and the concentrations of PFOS and PFOA in riverine sediments were in the range of <0.1 to 2.0 ng/g dw and <0.1 to 0.5 ng/g dw, respectively. In a coastal industrial area of Tianjin, concentrations of PFCs in surface waters and sediments ranged from 4.4 to 25 ng/L and 1.5 to 7.8 ng/g dw, respectively. All water samples contained detectable concentrations of PFOS and PFOA, while PFDoA and PFUnA were found to be ubiquitous in sediments. PFDoA ranged from 0.27 to 0.81 ng/g dw in sediment, with an average of 0.48 ng/g dw. In addition to direct input to Bohai Sea through surface runoff, precipitation events might be another source of PFCs. Liu et al. [25] analyzed PFCs in rain and snow samples in Dalian; they found a geometric mean 145 ng/L for PFOS and 24.7 ng/L for PFOA during the snow event. Ju et al. [26] measured PFOS and PFOA levels in Dalian coastal waters; the geometric means of PFOS and PFOA were 0.18 and 0.69 ng/L, respectively. Further, they found that PFCs concentrations in sea surface microlayer samples were significantly higher than surface water and subsurface water because of surfactant properties of PFCs.

### 3.3. Other Pollutants

Polychlorinated naphthalenes (PCNs) and HBCDs have been proposed as candidate compounds to the Convention recently. However, there are only a few reports about the pollution status of these emerging POPs in Bohai Sea. Pan et al. [27] analyzed PCNs in sediments from Laizhou Bay area. Concentrations of PCNs in marine and riverine sediments ranged from 0.06 to 0.47 ng/g dw and 0.05 to 5.1 ng/g dw, respectively. PCNs in marine sediments were slightly lower than those in Qingdao coast (located at the rim of an open ocean), and the latter ranged from 0.21 to 1.21 ng/g dw. The average concentration in riverine sediments was approximately 4 times higher than those in marine sediments (1.1 versus 0.26 ng/g dw), indicating the diffusion of PCNs from land to marine. Zhao et al. [28] investigated PCNs concentrations in sediments from the Daliao River Estuary. Total concentrations of PCNs in north Bohai Sea coastline were in the range of 0.03–0.28 ng/g dw, which were comparable to Laizhou Bay located in south Bohai Sea coastline [27]. Compared with other areas in China, sediment PCNs concentrations in Bohai Sea were similar to that in Yangtze River Estuary (0.03–0.30 ng/g dw), Qingdao coast (0.21–1.21 ng/g dw), and Yellow River Estuary (0.006–0.41 ng/g dw [29, 30]. Globally, PCNs in sediments were relatively low compared to North America [31], and the possible reason might be that commercial PCNs have never been historically produced in China. Zhang et al. have lately reported the concentrations of HBCDs in the sediment from Tianjin (Haihe River, Dagu Drainage Canal, and Tianjin Harbor), which filled the gap of HBCD study in this area [32]. The concentration of HBCD in the sediment turned out to be a very high value, which has reached 634 ng/g. HBCDs levels in riverine sediments in Laizhou Bay area, which is the biggest brominated chemical industry base in China, were reported by Li et al. [33]. The highest concentrations in sediment samples reached 1029 ng/g dw, which was obviously influenced by the production activities in local area.

## 4. Distribution of Emerging POPs in Biota

POPs can accumulate in the biota due to their hydrophobic and lipophilic characteristics and finally expose high health risk on higher-trophic-level organisms including humans *via* biomagnifications through food chains. The toxicities of emerging organic pollutants were not clear at present stage, and the levels of these pollutants in biota especially in edible organisms should be carefully concerned. Aquatic organisms such as mollusks and fishes have been proved to be good pollutants and risk indicators. They can reflect the contamination level in their surrounding environment such as water and sediment and also can evaluate the health risk on humans who intake the pollutants through seafood consumption [34].

### 4.1. PBDEs

Wang et al. [35] measured the levels, profiles, and distribution of PBDEs in mollusks of Bohai Sea from 2006 to 2007. PBDEs could be detected in all samples, which indicated the PBDEs' ubiquity in environment. The sum of 12 PBDEs (BDE17, 28, 47, 66, 71, 85, 99, 100, 138, 153, 154, and 183) ranged from 0.11 to 61 ng/g dw in 2006 and from 0.04 to 4.4 ng/g in 2007. In addition, PBDEs in mollusk samples collected from southern coastline were higher than those from northern coastline, which might be due to the disseminated BFR manufacturing industries on the southern coastline. In the recent study by the same group [36], the PBDEs in 131 mollusk samples collected in 2009 and 2010 were analyzed, and the concentrations ranged from 0.01 to 20.4 ng/g dw with a mean of 1.39 ng/g dw. They found that PBDE in mollusks decreased from 7.0 ng/g dw in 2003 to 1.0 ng/g dw in 2010. The downward trends might be due to the phasing out of penta- and octa-BDEs commercial products on a global scale and the restriction of PBDEs in China since 2006. Jin et al. [37] reported that PBDEs levels in four species of shellfish from Laizhou Bay ranged from 230 to 720 ng/g lipid weight. Wan et al. [38] analyzed 13 PBDEs congeners in different trophic levels (including zooplankton, invertebrate species, fishes, and marine birds) in Bohai Sea ecosystem. Total PBDEs contents in biota from Bohai Bay ranged from 0.15 to 32.8 ng/g lipid. BDE 47 was the predominant congener in most samples. Furthermore, biomagnifcation of PBDE in the marine food web is confirmed by stable nitrogen isotope technologies in the samples from Chinese Bohai Bay. Liu et al. [39] determined the levels of PBDEs in marine fish from all the four seas of China and the average of total PBDEs (exclude BDE 209) and BDE 209 concentration in fish from Bohai Sea was 24 and 12 ng/g wet weight (ww), respectively, which was lower than that in Yellow Sea and East China Sea. But PBDE contents in fish were relatively higher compared with those in mollusk samples [14, 35–37]. Another study on PBDEs in invertebrate and fish species collected from Bohai Bay from 2007 to 2008 [40] indicated that the concentrations of the six major PBDE congeners (BDE 28, 47, 99, 100, 153, 154) in fish were significantly higher than in invertebrates, which also proved the biomagnification of PBDEs in marine food web. Different from sediments, BDE 209 was not the predominant congener in biota samples and even below detection limits in some samples. This is consistent with the basic fact of the lower bioavailability and chemical stability of BDE 209. One exception is reported by Wang et al. [14], who found that BDE 209 was the major congener in mussel samples from Bohai Sea and the median of BDE 209 in mussels was about 4 times higher than that in sediments (2.43 versus 0.68 ng/g dw).

### 4.2. PFCs

The presence of PFCs was reported in biota samples from Bohai Sea, while PFOS and PFOA were major PFCs in biota. Pan et al. [41] first reported nine PFC compounds' levels in eleven mollusk species (soft tissues) which were collected from nine coastal cities along the Chinese Bohai Sea. PFOA was the predominant PFCs, followed by PFOS in 137 mollusk samples. Mean concentrations of PFOA and PFOS in different species ranged from <0.5 to 31.3 and 0.22 to 1.24 ng/g dw, individually. The concentrations of PFOA in mollusks were significantly correlated with those of PFOS, which indicated a common source of the two compounds in this region. Chen et al. [42] reported that the mean concentration of PFOS (1.78 ng/g ww) in wild marine crabs from Hangu (Tianjin) was slightly higher than that in mollusks. Yang et al. [43] measured 10 PFCs in seafood collected from Bohai Bay, including 8 invertebrate species and 6 fishes. Organ distribution indicated that PFCs were preferentially distributed in the liver or viscera in seafood. Total PFCs in invertebrates and fishes ranged from nd to 194 ng/g dw and 4.0 to 304 ng/g, respectively. Concentrations of PFCs in fish were greater than those in other species of lower trophic levels such as mollusks and invertebrates, which might suggest biomagnification process of PFCs in the food web of Bohai Sea.

### 4.3. Other Pollutants

Recently, not only the PFCs and PBDEs but also several other emerging POPs in mollusk samples from Bohai Sea were investigated. It included several types of BFCs and SCCPs. Zhu et al. [36] analyzed HBCDs and a novel BFC, tris(2,3-dibromopropyl) isocyanurate (TBC), in nine bivalve and two gastropod species collected from nine coastal cities around the Bohai Sea in 2009 and 2010. Concentrations of HBCDs and TBC ranged from nd to 28.8 ng/g dw and nd to 12.1 ng/g dw. The detection frequencies of HBCDs and TBC reached 99% and 77% in mollusks, indicating wide distribution of these two emerging POPs in Bohai Sea region. Yuan et al. [44] examined SCCPs contents in the same batch of mollusk samples and evaluated the spatial distributions and potential factors influencing the bioaccumulation. Concentrations of total SCCPs were in the range 64.9~5510 ng/g dw in the mollusks. Six and seven chlorinated substituents were the main congener groups in mollusks, with an average chlorine content of 61.1%. According to the carbon chain length, SCCPs with 10 and 11 carbon atoms were the predominant homologue groups, accounting for about 29.7% and 34.9% of the total SCCPs, respectively. *Mya arenaria, Mactra veneriformis*, and oyster could be selected as potential bioindicators for investigating SCCPs contamination in the coastal region.

## 5. Comparison of Emerging Organic Pollutants between Bohai Sea and Worldwide

**Figure 2:**
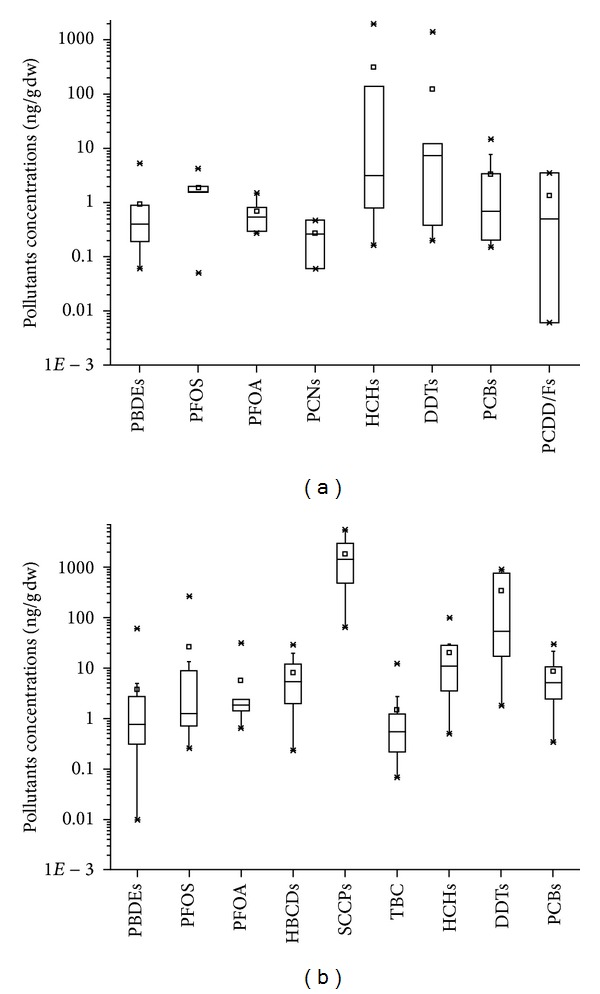
(a) Legacy POPs and emerging pollutants in Bohai Sea sediments. The upper and lower quartiles of the boxes represent 25th and 75th percentiles of the data, and three horizontal bars represent the maximum, median, and minimum values; “×” and “*▫*” denotes outliers and mean. (Data source: PBDEs [14, 15, 17, 18, 20]; PFOS [23, 24]; PFOA [23, 24]; PCNs [27]; HCHs [6, 84, 85]; DDTs [6, 85–88]; PCBs [15, 19, 87, 89]; PCDD/Fs [84, 89]). (b) Legacy POPs and emerging pollutants in Bohai Sea organisms. The upper and lower quartiles of the boxes represent 25th and 75th percentiles of the data, and three horizontal bars represent the maximum, median, and minimum values; “×” and “*▫*” denotes outliers and mean. (Data source: PBDEs [14, 35–37, 40]; PFOS [23, 41]; PFOA [23, 41]; HBCDs [36]; SCCPs [44]; TBC [36]; HCHs [9, 35, 37]; DDTs [9, 52]; PCBs [11, 35]).

The pollution status of both legacy and emerging pollutants in the Bohai Sea area has been summarized in Figures 2(a) and 2(b). Compared to legacy POPs, levels of emerging pollutants (PBDEs, PFOS, PFOA, and PCNs) in sediments were lower than organochorine pesticides (HCHs and DDTs) but were comparable to PCBs and PCDD/Fs (Figure 2(a)).  Figure 2(b) showed the status of pollutants in biota samples from Bohai Sea, among which, the level of SCCPs was the highest, followed by the order of organochorine pesticides (DDTs, HCHs) > PCBs, PFOS, HBCDs > PFOA, PBDEs, TBC. The PBDE concentrations in sediments and organism in the Bohai Sea were comparable to those from other sea areas of China. PFCs levels in sediments from Bohai Sea also showed similarity to other sea areas in China. But in biota samples, PFCs levels were slightly higher in Bohai Sea, which might be due to its semiclosed topography and the contribution of several local rivers intensively affected by human activities [22]. Emerging persistent pollutants levels in developed countries' coast (such as USA and Japan) and remote areas (such as north Norway, Greenland Island, and Canada) were also listed to make a comparison to the Bohai Sea (Table 1). On the whole, PBDEs levels in samples from Bohai Sea area were lower than those from North America, which might be due to the large usage amount of PBDEs in the continent [45]. Even at the most contaminated sites in Bohai Sea area (Laizhou Bay) [17], PBDEs level in sediments were still lower than that in the USA [46, 47]. PFOS and PFOA in Bohai Sea area were comparable to foreign contamination level [46, 47] except those in Arctic area [48].

## 6. Conclusions and Perspectives

Emerging POPs have aroused wide public concern in recent years. The present study reviewed the status and congener profiles of emerging organic pollutants in the Chinese Bohai Sea, which is surrounded by several metropolises. Surprisingly, the pollution levels of some kinds of emerging organic pollutants (PFOS, HBCDs) in organisms were comparable to some legacy POPs, although the former has been paid attention to recently. Besides, the emerging pollutants also exhibited ubiquitous existence in both abiotic and biotic media. Such results suggested that the emerging organic pollutants in Bohai Sea should arouse our attention. Moreover, some of these pollutants are bioaccumulative and biomagnified through food web and finally can expose high risk to human beings. Though the data in Figure 2 come from different studies, they have shown some consistency: the average values of PBDEs, PFOS, and PFOA were higher in organism than those in sediments, which indicated bioaccumulation behavior of these emerging chemicals (organism versus sediment: 3.85 versus 0.94 for PBDEs, 26.35 versus 1.90 for PFOS, 5.72 versus 0.68 for PFOA). Studies on the occurrence and environmental behavior of organic pollutants in Bohai Sea increased dramatically in recent years, while the pollution profiles, levels, temporal trends, and transport mechanism of emerging organic pollutants in this area are still very limited. The data for some emerging chemicals such as TBBPA, a common brominated flame retardant, is not available in this area in spite of the fact that TBBPA has been found in other water areas [49, 50] and even Arctic area [51]. HBCD acted as a candidate chemical of Stockholm Convention now, but there are very limited data reported in this area. Up to now, only a few studies provided the pollution levels in water and marine sediment samples directly obtained in Bohai Sea. The possible reason may be (1) the low levels of these pollutants in water samples and (2) the difficulties to collect marine sediments. On the contrary, biological samples have been used extensively to assess the organic pollutant levels, source identification, biotransformation, and temporal trends due to the relative higher concentrations and easier collection [35, 36, 52–54]. Certain biota (especially mussels) have been used as indicator for monitoring emerging organic pollutants in the Bohai Sea, which allowed us to compare the status of emerging organic pollution among different countries and regions. Bohai Sea is a relative closed ecological system, which make it an ideal place for investigating the trophodynamic behavior of organic pollutants [55, 56]. In present stage, few studies were related to emerging pollutants transportation along food web in this area, whereas the bioaccumulation and biomagnifications of these emerging organic pollutants were still not clear. As for temporal issue, Zhu et al. [36] reported a decline trend of PBDEs in mollusks during 2003 to 2010 (from 7.0 to 1.0 ng/g dw). However, there were no data available to evaluate the temporal trends for other emerging pollutants in this area. Thus it is necessary to select certain bioindicators from Bohai Sea to set up a long term monitoring program. Besides, spatial monitoring in the sea and along the coastal areas is also needed. With the GPS information and congener patterns from all the sampling sites, it is able to describe an “emerging POPs map system” in this special area, which would be very helpful to trace the pollution sources. The idea has already been used in legacy POPs [57]. Through the temporal and spatial trends of legacy and emerging pollutants, on one hand, we can track the changing pollutant profiles and adjust the management policy. On the other hand, it can also provide a data basis for the environmental safety of China and Stockholm Convention implementation.

## Figures and Tables

**Table 1:** Comparison of emerging organic pollutants in different matrix among Bohai Sea and other areas.

Location		PBDEs	PFOS	PFOA	HBCD	Sample matrix
China	Bohai Sea	0.07–5.24 [14] 0.22–0.9 [15] nd-0.66 [17] nd-0.87 [18] 0.13–1.98 [20]	nd-1.97 [23] nd-4.3 [24]	nd-0.54 [23] nd-1.5 [23]	—	Sediment
		0.31–2.73 [14] 0.04–60.9 [35] 0.01–20.4 [36]	nd-4.33 [41] nd-268 (Ark shell 0.94) [43]	nd-126 [41] nd-60.6 (Ark shell 2.10) [43]	nd-28.8 [36]	Biota

China	Other sea areas	0.1–5.5 [29] nd-8.0 [58] 0.04–4.48 [59]	<0.1–11.91 [60]	1.84–34.01 [55]	—	Sediment
		Nd-2.20 [61] 0.306 (mussel, average) (ww) [62] 84–980 (small cetaceans) (lw) [63]	0.3–13.9 (ww) [64] nd-1.69 (ww) [65] <0.0014–1.627 (ww) [66] 0.6–2.0 [67]	nd-1.67 (ww) [59] nd-0.66 (ww) [60] <0.0054–7.543 (ww) [61] nd-2.2 [47]	4.7–380 (small cetaceans) (lw) [63] nd-51 (lw) [68]	Biota

Japan		0.05–3.6 [69]	0.09–0.14 [70] 0.13–1.4 [71]	0.84–1.1 [70] 0.11–0.39 [71]	0.056–2.3 [69]	Sediment
		1.3–8.5 (lw) [72] 0.03–2.0 (ww) [73]	<0.3 [70] 3.8 [67]	6.3–14 (mussel) (ww) [70] 3.4–8.1 (oyster) (ww) [70] 4.3 [67]	0–36.9 (ww) [74] 6.2–49 (lw) [34] <0.5–110 (lw) [72] 0.3–10 (ww) [73] 10–1400 (lw) [68] 44.3 (black-striped mussel)–177 (green crab) (lw) [75]	Biota

USA		nd-212 [47] 2.1–8 [7] nd-560 [46]	0.07–0.18 [76]	0.03–0.08 [76]	0.1–1.7 [77]	Sediment
		9–106 [47] 470–2260 (White croaker) (lw) [77] 30.4–4500 (Bottlenose dolphin) (lw) [78] 12.4–4190 (Bull shark) (lw)[78]	1.4–96.8 (ng/mL Sea turtle Plasma) [79] 0.31–39.0 (ng/mL Sea turtle Plasma) [80]	0.49–8.14 (ng/mL Sea turtle Plasma) [79] 0.076–0.993 (Sea turtle Plasma) [80]	nd-4.5 (White croaker) (lw) [77] 0.460–72.6 (Bottlenose dolphin) (lw) [78] 9.15–413 (Bull shark) (lw) [78]	Biota

Arctic	Area	0.107–0.297 [51]	<0.04^82^ nd-0.11 [48]	0.017–0.13^82^ nd [48]	nd [51]	Sediment
		0.11 (ww) [81] 0.06–0.19 (ww) [51] 18.3–44.5 (eggs of ivory gulls) (lw) [82]	0.12–5.4 (Cod muscle) (ww) [83] <0.3–1.3 (Salmon muscle) (ww) [83]	0.14–0.03 (Cod muscle) (ww) [83] <0.08–1.2 (Salmon muscle) (ww) [83]	14.7 (Polar Cod) (lw) [51] 2.1–3.8 (eggs of ivory gulls) (lw) [82]	Biota

(ng/g dw ww: wet weight based; lw: lipid weight based).
